# Reporting quality of systematic reviews published from 2011 to 2024 in the *Journal of Korean Biological Nursing Science* based on the PRISMA 2020 checklist: a methodological review

**DOI:** 10.7586/jkbns.25.078

**Published:** 2026-02-23

**Authors:** Mi-Kyoung Cho, Mi Young Kim

**Affiliations:** 1 College of Nursing Science, Chungbuk National University, Cheongju, Korea; 2 Department of Nursing Science, College of Nursing, Chungbuk National University, Cheongju, Korea

**Keywords:** Systematic reviews as topic, Nursing research, Guideline adherence, Quality control

## Abstract

**Purpose:**

This study evaluated the reporting quality of systematic reviews (SRs) published in the *Journal of Korean Biological Nursing Science* (JKBNS) from 2011 to 2024 using the Preferred Reporting Items for Systematic Reviews and Meta-Analyses (PRISMA) 2020 guideline and examined conceptual alignment with A Measurement Tool to Assess Systematic Reviews 2 (AMSTAR-2) and Risk of Bias in Systematic Reviews (ROBIS).

**Methods:**

A methodological review was conducted using articles retrieved from the JKBNS archive. The literature search was performed between October 7 and October 24, 2025. Two reviewers independently screened all records, and reporting quality was assessed using the 27 PRISMA items and 42 associated sub-items, which were scored as fully reported (1), partially reported (0.5), or not reported (0). Compliance rates were calculated, and PRISMA items were mapped to corresponding AMSTAR-2 and ROBIS domains.

**Results:**

Twenty SRs and meta-analyses met the inclusion criteria. The mean score across PRISMA sub-items was 23.58 ± 7.34 (range, 15~38). Ten sub-items—including rationale, objectives, information sources, search strategy, data collection, study selection and exclusion, study characteristics, and key discussion elements—were consistently reported. In contrast, protocol registration, protocol access, and sensitivity analyses were rarely reported, while certainty assessment, certainty of evidence, and protocol amendments were not reported in any study. Overall adherence to the 27 PRISMA items was 64.7 ± 14.9% (range, 44.4~92.6).

**Conclusion:**

Conceptual mapping demonstrated strong alignment between PRISMA methodology and results items and the domains of AMSTAR-2 and ROBIS; however, transparency-focused components newly introduced in PRISMA 2020 showed limited correspondence. These findings provide practical guidance for improving reporting transparency in future SRs published in JKBNS.

## INTRODUCTION

### 1. Background

Systematic reviews (SRs) represent a research design central to evidence-based nursing practice, playing a critical role in comprehensively evaluating the effectiveness and safety of nursing interventions. The quality of a SRs is influenced not only by the quality of the primary studies but also by the transparency and completeness of its reporting [1]. Insufficient or unclear reporting can reduce the reproducibility of the research and may provide misleading evidence for clinical practice or policy-making [2]. Therefore, SRs require reporting in accordance with clear and standardized guidelines.

To standardize the reporting of SRs, the Preferred Reporting Items for SRs and Meta-Analyses (PRISMA) guideline has been widely adopted internationally. In 2020, PRISMA 2020 was released to address the limitations of the previous 2009 version [2]. The updated version emphasizes greater specificity in key elements, including the transparency of search strategies, the processes for study selection and data extraction, methods for assessing risk of bias, and the evaluation of the certainty of evidence [3].

Despite the rapid evolution of international standards and the increasingly stringent reporting requirements, adherence to these updated guidelines varies across journals and specific disciplines. Studies have shown that, in some fields, SRs are not consistently reported in accordance with PRISMA 2020 [4]. Moreover, overall compliance with PRISMA 2020 has been reported to be low, with substantial variability across individual checklist items [5]. Consequently, it is necessary for each journal to assess the specific adherence status of published reviews. In particular, items introduced in the latest version, such as the assessment of certainty of evidence and prospective protocol registration, are likely to have lower compliance rates. Such deficiencies may compromise both the clinical utility and the scientific credibility of SRs published in the journal.

In the nursing field in Korea, the number of SRs publications has been steadily increasing; however, research assessing the consistency and completeness of their reporting remains limited. The *Journal of Korean Biological Nursing Science* (JKBNS), a leading journal encompassing nursing interventions, physiological studies, and educational research, plays a pivotal role in disseminating high-quality evidence. The reporting quality of SRs published in this journal has a significant impact on both academic credibility and clinical applicability. Nonetheless, a comprehensive analysis of the extent to which SRs in JKBNS adhere to PRISMA 2020 guidelines has not yet been conducted. Therefore, this study aims to evaluate the reporting quality of SRs published in JKBNS from 2011 to 2024 based on the PRISMA 2020 checklist, providing foundational data to enhance reporting standards in the nursing field.

This study aims to comprehensively analyze the level of adherence to PRISMA 2020 guidelines, identify key reporting deficiencies, and examine the alignment with international quality assessment tools such as A Measurement Tool to Assess Systematic Reviews 2 (AMSTAR-2) and the Risk of Bias in Systematic Reviews (ROBIS). While the use of PRISMA 2020 ensures the completeness of reporting, it does not fully assess the methodological quality or the reliability of results considering the review process itself [6]. AMSTAR-2 evaluates whether the design, conduct, and analysis of a review conform to international standards, whereas ROBIS provides a structured assessment of the risk of bias in SRs, thereby enhancing the suitability of findings for recommendations and policy-making [7]. Therefore, this study allows for a comprehensive evaluation of both reporting quality and methodological rigor.

Therefore, this study aims to evaluate the reporting quality of SRs and meta-analyses published in JKBNS from 2011 to 2024 using the PRISMA 2020 checklist. The analysis further integrates the alignment between reporting guidelines and quality assessment tools, providing evidence-based recommendations for authors, reviewers, and editorial boards. By comprehensively examining these aspects, the study seeks to empirically identify the current status and areas for improvement in the reporting quality of SRs in the Korean nursing field, and to offer practical guidance for conducting and reporting SRs in accordance with international standards in the future.

### 2. Study aim

This study aimed to evaluate the reporting quality of SRs published in the JKBNS from 2011 to 2024, based on the PRISMA 2020 reporting guidelines. The findings are intended to inform authors, peer reviewers, and journal editors about current reporting practices and to provide guidance for enhancing the methodological transparency of SRs in JKBNS. The specific objectives of the study were to: summarize the general characteristics of SRs published in JKBNS during the study period; assess item-level compliance with each element of the PRISMA 2020 checklist; and map PRISMA 2020 checklist items to corresponding components in AMSTAR-2 and ROBIS tools.

## METHODS

### 1. Study design

The design of this study was a methodological review aimed at evaluating the reporting quality and methodological transparency of SRs published in JKBNS from 2011 to 2024, rather than synthesizing clinical evidence.

### 2. Data source and search strategy

The data source for this study was the official online archive of the JKBNS (https://jkbns.org/articles/archive.php). The search was conducted between October 7 and October 24, 2025. A comprehensive list of articles published between 2011 and 2024 was compiled using Microsoft Excel (Office 365; Microsoft Corporation, Redmond, WA, USA), including bibliographic information such as authors, year of publication, article title, DOI, and study design. In the first screening phase, article titles and abstracts were searched using the following keywords: 'review', 'systematic review', and 'meta-analysis'. This search yielded 37 potentially relevant articles from a total of 479 publications.

### 3. Eligibility criteria

### Studies were eligible for inclusion if they met the following criteria:

**Figure 1.**
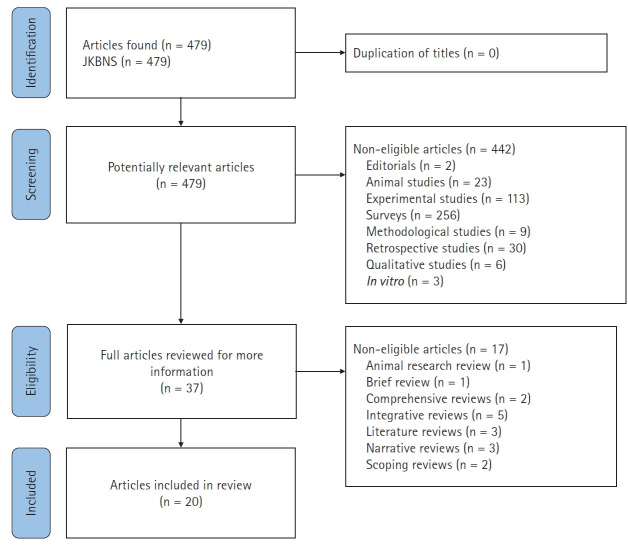
Flow chart of study selection. JKBNS = *Journal of Korean Biological Nursing Science*.

(1) published in JKBNS between 2011 and 2024; and (2) explicitly identified as a SRs or meta-analysis in the title, abstract, or methods section. The exclusion criteria were as follows: (1) non-SRs designs, including literature reviews, integrative reviews, comprehensive reviews, and brief reviews; (2) scoping reviews, which do not align with the PRISMA 2020 framework; (3) narrative reviews; (4) animal-based intervention reviews. The study selection process is shown in Figure 1.

### 4. Instruments

#### 1) PRISMA 2020 checklist

The PRISMA statement, developed by Moher et al. [8], is an internationally recognized reporting guideline designed to improve the quality of reporting in SRs and meta-analyses. The guideline originated with the Quality of Reporting of Meta-analyses (QUOROM) statement, first introduced in 1999 to improve the completeness and transparency of meta-analysis reports [9]. In 2009, PRISMA was published as a revised version of QUOROM, introducing more precise definitions of research questions and a standardized flow diagram to enhance transparency in reporting [9]. Later, Page et al. [2] updated the guideline to emphasize research transparency, reproducibility, and data sharing in response to the evolving research environment and open science practices. Compared with the 2009 version, PRISMA 2020 has been substantially updated. The revision included a dedicated checklist for abstracts (PRISMA for abstracts), an expanded scope of information sources, including registers, websites, and automated tools, and a clearer presentation of study exclusion reasons, with a more detailed flow diagram. In addition, the updated version required explicit reporting of the risk-of-bias assessment tools, number and independence of reviewers, and use of automation.

Certainty or confidence in evidence, such as Grading of Recommendations, Assessment, Development, and Evaluation (GRADE), became a mandatory item, and new elements addressing data, code, and analytic transparency were also incorporated. These changes reflected the increasing expectations for reproducibility and open access to data in health research. The PRISMA 2020 checklist consists of seven sections comprising 27 main items and 42 sub-items.

The title and abstract domain requires authors to identify the report as SRs in the title and to summarize the methods and results in accordance with the PRISMA for abstracts checklist. The introduction domain recommends that authors clearly describe the rationale and objectives of the review. The methods domain covers the eligibility criteria, search strategy, selection process, data collection process, risk-of-bias assessment, effect measures, synthesis methods, reporting bias, and certainty assessment. The results domain includes the presentation of study selection, characteristics, risk-of-bias results, synthesis findings, reporting bias, and certainty of evidence. The discussion domain guides authors to interpret key findings, discuss limitations in both the included studies and the review process, and highlight implications for practice, research, and education. Finally, the other Information domain recommends reporting registration details, protocol accessibility, funding, conflicts of interest, and data availability.

The evaluation of PRISMA 2020 checklist adherence is based on the completeness of reporting for each checklist item. Each item can be rated as ‘Yes’ when the requirement is fully met, ‘Partial’ when information is incomplete or unclear, or ‘No’ when the item is not reported [10]. In this study, ‘yes, partial, and no’ were scored as 1, 0.5, and 0 points, respectively. Higher total scores indicate greater transparency and reproducibility of reporting, and the PRISMA adherence rate (i.e., the proportion of adequately reported items among the 27 items) can be used to compare the reporting quality across SRs. However, the PRISMA 2020 checklist assesses reporting quality rather than methodological quality; therefore, it is recommended that it be used together with methodological appraisal tools such as AMSTAR-2 or ROBIS for a comprehensive evaluation of SRs [11,12].

#### 2) AMSTAR-2

AMSTAR-2 is a validated critical appraisal tool developed by Shea et al. [11] to assess the methodological quality of SRs that include randomized or non-randomized studies of healthcare interventions. This tool represents a significant revision of the original AMSTAR instrument, introduced in 2007 and previously restricted mainly to reviews of randomized controlled trials [12], thereby allowing the evaluation of both randomized trials and quasi-experimental designs [11]. AMSTAR-2 consists of 16 items, each of which evaluates a key methodological domain relevant to the design and conduct of SRs [13]. These domains include defining the research question and inclusion criteria based on a population, intervention, comparison, outcome (PICO) framework, adequacy of the literature search, justification for the selection of study designs, duplicate study selection and data extraction, description of included studies, assessment of risk of bias, appropriateness of meta-analytic methods, consideration of risk of bias when interpreting results, assessment of publication bias, and declaration of conflicts of interest. Each item is rated as ‘yes, partial yes, or no,’ depending on whether the methodological standard is fully met, partially met, or not met.

Among the 16 items, seven are considered to have the most significant influence on the overall confidence in the review’s findings. These critical items include protocol registration (item 2), adequacy of the literature search (item 4), justification for excluding individual studies (item 7), risk-of-bias assessment of individual studies (item 9), appropriateness of meta-analytic methods (item 11), consideration of risk of bias when interpreting results (item 13), and assessment of publication bias (item 15).

#### 3) ROBIS

ROBIS, developed in 2016 by Whiting et al. [14], is a validated tool for assessing risk of bias in SRs and is implemented in three phases. Phase 1, conducted as needed, assesses the relevance of the review and helps users determine whether the population, intervention, comparison, outcomes, and study design (PICOS) are appropriately aligned with the review’s intended objectives [14,15]. Phase 2 serves as the core assessment stage and consists of four domains that evaluate potential sources of bias: study eligibility criteria; study identification and selection; data collection and study appraisal; and synthesis and findings. Each domain includes signaling questions designed to guide reviewers in determining concerns related to the methods or reporting of the review, and each signaling question is typically answered using the categories ‘yes, probably yes, probably no, no, or no information’ [15]. Each domain is subsequently assigned an overall level of concern—'low, high, or unclear’ — based on the reviewer’s judgment [13,14]. Phase 3 synthesizes the judgments from Phase 2 to determine the overall risk-of-bias rating for the review, which is classified as ‘low, high, or unclear’, depending on the extent of concerns identified across domains [14,16].

### 5. Data collection

The data for the included studies were retrieved from the official online archive of the JKBNS and organized in a data extraction spreadsheet created using Microsoft Excel (Office 365; Microsoft Corporation). Reasons for exclusion at both the first and second screening stages were documented within this spreadsheet. Bibliographic information, including author(s), publication year, article title, DOI, and study design, was compiled for all articles published between 2011 and 2024. To ensure accurate classification of study designs, studies identified as SRs or meta-analyses based on their titles, abstracts, or methodological descriptions were independently assessed by two reviewers (Cho & Kim) using predefined eligibility criteria. In the first screening stage, studies were excluded after reviewing the title and abstract, whereas in the second stage, exclusions were made following full-text assessment. The reviewers jointly discussed each step of the selection process to determine final inclusion.

Disagreements primarily involved decisions regarding the inclusion of literature reviews, integrative reviews, comprehensive reviews, and narrative reviews. These cases were resolved through discussion, and it was agreed that these types of studies would be added to the exclusion criteria and excluded accordingly. Additionally, the reviewers reached consensus that PRISMA items 15 (certainty assessment) and 22 (certainty of evidence) would be considered ‘reported’ only when the study explicitly described evidence grading or certainty appraisal process (e.g., GRADE or an equivalent framework). All decisions related to study selection and classification were documented to ensure methodological transparency.

Meanwhile, to ensure conceptual coherence across PRISMA 2020, AMSTAR-2, and ROBIS, the research team undertook a structured conceptual mapping procedure. Each PRISMA 2020 item was independently reviewed by all evaluators and compared with the corresponding domains and constructs in AMSTAR-2 and ROBIS. Given the research team’s extensive experience in conducting and appraising SRs, the evaluators shared a common understanding of the conceptual boundaries and intent of each item. As a result, complete agreement was achieved across all mappings, and no discrepancies requiring adjudication or consensus meetings were identified.

### 6. Data analysis

Data analysis was conducted to summarize the general characteristics of the included SRs and meta-analyses and to evaluate the completeness of reporting according to the PRISMA 2020 reporting guideline. A conceptual comparison was also performed to examine areas of alignment and divergence across PRISMA 2020, AMSTAR-2, and ROBIS. The characteristics of the included studies were extracted for the following categories: (1) bibliographic information (author, publication year, study topic or aim); (2) methodological characteristics (PICO reporting, protocol registration and registry source, number and types of databases searched, number of reviewers involved in study selection and assessment, use of a flow diagram, and number of included studies); (3) quantitative synthesis characteristics (effect size reporting, methods of assessing heterogeneity, and approaches to evaluating publication bias); and (4) risk-of-bias or methodological quality assessment tools used. All extracted information was coded using a structured Excel coding book.

Reporting quality was assessed using the PRISMA 2020 checklist comprising 7 domains, 27 items, and 42 sub-items. Each sub-item was coded as ‘fully reported’ (1 point), ‘partially reported’ (0.5 points), or ‘not reported’ (0 points), and total PRISMA scores (range: 0~42) were calculated for each study. Compliance scores for the 27 PRISMA items were obtained by summing the corresponding sub-item scores and recoding each item as 1 (fully reported), 0.5 (partially reported), or 0 (not reported). For item 23, reporting was considered complete if limitations related to either the included evidence or the review process were described, consistent with PRISMA 2020 guidance. For item 24, studies were not penalized for failing to report protocol amendments; therefore, the item was scored as fully reported when protocol registration information was provided, even if amendments were not applicable. The recoded item-level scores were then summed to yield a total item score ranging from 0 to 27, and overall adherence was expressed as a percentage of the maximum possible score. To contextualize reporting completeness, PRISMA 2020 items were then conceptually mapped to the corresponding domains of AMSTAR-2 and ROBIS to compare convergence and conceptual distinctions between reporting guidelines and methodological quality assessment tools.

### 7. Ethical considerations

This review was based entirely on data extracted from previously published studies and did not involve contact with human participants or the collection of new individual-level information. As such, the study did not require Institutional Review Board approval under prevailing ethical guidelines for secondary analyses of publicly accessible data. All information drawn from the included articles was accurately cited to ensure appropriate acknowledgment of sources and to maintain integrity in scholarly reporting. To further minimize potential bias, study selection and data extraction were independently conducted by two reviewers.

## RESULTS

### 1. Overview of SRs published from 2011 to 2024

The characteristics of the included SRs are summarized in Table 1. The number of SRs publications has increased over time, with 7 (35.0%) published between 2021 and 2024. Among the included SRs, 13 (65.0%) were SRs without meta-analysis, while 7 (35.0%) conducted a meta-analysis. Regarding the use of the PICO framework, 10 (50.0%) of the studies applied it, whereas only 2 (10.0%) reported protocol registration in the International Prospective Register of Systematic Reviews (PROSPERO, https://www.crd.york.ac.uk/prospero).

The number of databases searched ranged from 2 to 12, with a mean of 6.40 ± 3.28. Nine studies (45.0%) searched fewer than six databases. Among international databases, PubMed was the most frequently used (n = 15, 75.0%), followed by Cumulative Index to Nursing and Allied Health Literature (CINAHL) (n = 14, 70.0%), the Cochrane Library (n = 10, 50.0%), and Embase (n = 10, 50.0%). For Korean databases, the Research Information Sharing Service (RISS) was most frequently used (n = 17, 85.0%), followed by the Korean studies Information Service System (KISS) (n = 9, 45.0%) and the Korea Medical Database (KMbase) (n = 8, 40.0%). Regarding data collection, 17 studies (85.0%) involved two or more reviewers. A total of 12 studies (60.0%) included more than 10 primary studies, with a mean of 14.80 ± 10.89 (range: 5~53). Among the seven studies that conducted meta-analyses, four reported effect sizes using Hedges’ g. Heterogeneity was assessed in three studies (15.0%) using Cochran’s Q statistic and in 7 (35.0%) using Higgins’ I². Publication bias was evaluated in six studies (30.0%) using funnel plots. For quality appraisal, seven studies (35.0%) used the Risk of Bias (RoB) or Risk of Bias Assessment tool for Non-randomized Studies (RoBANS) tools, followed by the Joanna Briggs Institute (JBI) checklist (n = 3, 15.0%), the Newcastle-Ottawa Scale (NOS) (n = 2, 10.0%), the Critical Appraisal Skills Programme (CASP) (n = 1, 5.0%), and the Critical Review Form (n = 1, 5.0%).

### 2. Assessment of reporting quality based on the PRISMA 2020 checklist

Based on the 42 PRISMA 2020 checklist sub-items (Table 2), the included studies demonstrated an overall mean reporting score of 23.58 ± 7.34 (range: 15~38). Ten sub-items—3 (describe the rationale), 4 (objectives of the review), 6 (information sources),7 (search strategy), 9 (data collection process), 16a (study selection results), 16b (study selection exclusions), 17 (study characteristics), 23a (interpretation of results), and 23d (implications for practice, policy, and research)—were fully reported in all 20 studies (Appendix 1). Items 5 (eligibility criteria) and 8 (selection process) were fully reported in 19 studies, while the remaining two studies provided only partial reporting. At the lower end of the reporting performance scale, items 24a (registration information) and 24b (protocol access) were reported in only two studies. In addition, Items 13f (synthesis methods for sensitivity analysis) and 20d (sensitivity analysis results) were reported in only three studies. In contrast, items 15 (certainty assessment), 22 (certainty of evidence), and 24c (protocol amendments) were not reported in any study.

The mean adherence rate to the PRISMA 2020 checklist across the included studies was 64.7% ± 14.9% (range: 44.4~92.6), and overall, the SRs and meta-analyses (Study ID: 18~20) demonstrated a generally high level of reporting compliance (Table 3).

### 3. Mapping PRISMA 2020 checklist items to AMSTAR-2 and ROBIS

In this study, conceptual mapping was conducted using the PRISMA 2020 checklist as the reference framework to examine the alignment between reporting guideline items and the methodological quality criteria assessed by AMSTAR-2 and ROBIS (Table 4). Whereas PRISMA 2020 provides an international standard for reporting completeness in SRs and meta-analyses, AMSTAR-2 and ROBIS were developed to evaluate the methodological rigor and risk of bias of SR processes. Despite these fundamental differences in purpose, the mapping revealed several areas of conceptual convergence across the tools.

First, the introduction and methods components of PRISMA 2020 (items 3~8) showed strong alignment with key AMSTAR-2 (items 1, 3, 4, 5, and 8) and with ROBIS domains 1 and 2. This convergence reflects the shared emphasis across the three tools on ensuring research quality through clearly defined research questions, appropriate search strategies, and transparent study selection processes. For example, PRISMA items 5 (eligibility criteria), 6 (information sources), and 7 (search strategy) correspond closely to AMSTAR-2 items dealing with literature search, study selection, and exclusion procedures (items 1, 3, 4, and 8), as well as to ROBIS domain 1 (study eligibility criteria) and domain 2 (identification and selection of studies). These findings indicate that both reporting guidelines (PRISMA) and quality appraisal tools (AMSTAR-2, ROBIS) consider methodological transparency essential and require its reporting in SRs manuscripts.

Furthermore, PRISMA 2020 items 9~15, which relate to data collection, risk-of-bias assessment, and synthesis methods, mapped broadly to AMSTAR-2 core (items 6, 8, 9, 11, 13, and 15) and ROBIS domains 3 and 4. Likewise, the PRISMA results section (items 16~22) showed extensive correspondence with AMSTAR-2 (items 7, 8, 9, 11, 12, 13, 14, and 15) and ROBIS domains 2~4. These areas of overlap demonstrate that, when assessing the reliability of SRs findings, all three tools emphasize key methodological components, including assessing individual study bias, evaluating the appropriateness of synthesis methods, evaluating heterogeneity, and identifying publication bias.

In contrast, PRISMA 2020 items related to the discussion section (item 23) and several newly emphasized transparency components—specifically protocol registration and amendments (item 24), funding (item 25), and declarations of competing interests (item 26)—mapped onto corresponding AMSTAR-2 (items 2, 10, 13, 14, and 16), but showed no direct alignment with ROBIS. This divergence reflects that PRISMA 2020 and AMSTAR-2 incorporate more recent developments in research transparency, open science, and reproducibility. ROBIS remains focused on a more traditional framework for methodological risk-of-bias assessment. Additionally, PRISMA item 27 (availability of data, code, and other materials), newly emphasized in PRISMA 2020, had no corresponding item in either AMSTAR-2 or ROBIS. This indicates that PRISMA 2020 sets a broader and more stringent standard for transparency in SRs reporting than either of the two methodological appraisal tools.

## DISCUSSION

This study is a methodological review evaluating the reporting quality of SRs published in JKBNS from 2011 to 2024 based on the PRISMA 2020 checklist. The analysis revealed that several key items—such as the specificity of search strategies, explicit reporting of risk of bias assessments, and transparency of data handling and meta-analysis procedures—were relatively well reported. In contrast, items including protocol registration, clear description of data sources, and assessment of certainty of evidence (e.g., using GRADE) were generally reported inadequately. These findings suggest that, in order for SRs in the Korean nursing field to fully reflect current international reporting standards, ongoing education for researchers and strengthened editorial and peer-review policies at the journal level are warranted.

A detailed review of reporting indicators showed that the overall reporting quality of SRs published in JKBNS, as assessed in this methodological analysis, had an average adherence rate of 64.7%. The results of the item-level analysis are as follows. Reporting quality for key, traditionally emphasized steps in the SRs process was generally very high. Specifically, ten items—describing the rationale, objectives of the review, information sources, search strategy, data collection process, study selection results, study selection exclusions, study characteristics, interpretation of results, and implications for practice, policy, and research—were fully reported in all 20 studies. Additionally, eligibility criteria and the selection process were adequately reported in 19 studies, demonstrating a high level of adherence.

These findings suggest that authors submitting to JKBNS possess a sufficient understanding of the fundamental structure and core methodologies of SRs (e.g., PICO formulation, development of comprehensive search strategies) and, based on this understanding, report the basic procedures and results diligently. In other words, key elements required by international guidelines—such as the composition of search strategies, reporting of study selection and exclusion processes, and summary of characteristics of included studies—appear to be appropriately fulfilled.

First, regarding the assessment of item 2 in the PRISMA 2020 for Abstracts checklist, 12 out of 20 studies reported only some of the required elements partially. This is likely due to the relatively large number of reporting items expected in abstracts, combined with word count limitations imposed by most journals, making it difficult to include all items. Nevertheless, it is important for journal author guidelines to clearly specify the essential items that must be included, ensuring that critical information is not omitted even within the limited space of an abstract.

On the other hand, areas newly emphasized in PRISMA 2020, such as transparency and assessment of the certainty of evidence, were found to be inadequately reported. In particular, the item on certainty of evidence showed a 0% adherence rate, and transparency-related items regarding protocol registration and disclosure also demonstrated low compliance, ranging from 0% to 10%. These deficiencies represent major limitations that could undermine the clinical applicability and scientific credibility of SRs. Specifically, the findings indicate systematic reporting gaps in items highlighted as core elements in contemporary evidence-based practice and open science principles. Notably, methodological item 15 (Certainty assessment) and results item 22 (Certainty of evidence) were not adhered to in any of the studies. This suggests that many SRs, while focusing on the execution of meta-analyses, did not conduct essential steps such as evaluating the quality of evidence (e.g., using GRADE), which are crucial for clinical interpretation and practical application. The omission of such information may compromise the clinical utility and translational value of the evidence provided by these reviews [17]. Furthermore, it not only hinders the reproducibility of the research but fundamentally weakens the trustworthiness of the results. Certainty assessment, in particular, is a critical component that clarifies the level of confidence in study findings, enabling end users to determine the degree of assurance required when applying the evidence in clinical practice or policy decisions [18]. Therefore, enhancing the reporting of these items necessitates clearer author guidelines and active promotion of adherence.

Additionally, item 20d (Results of syntheses for sensitivity analyses), which is critical for assessing the robustness of results, was reported in only 3 of the 20 studies, representing a low adherence rate of approximately 15%. This finding aligns with previous research indicating that sensitivity analysis reporting is generally limited even within specialized fields [4]. Sensitivity analyses systematically evaluate the impact of methodological uncertainties—such as the inclusion of studies at high risk of bias or changes in statistical models—on key outcomes. The insufficient reporting of these analyses suggests that the effects of critical methodological decisions on the results were not adequately assessed. Consequently, the published meta-analytic findings may represent potentially fragile conclusions [19]. Therefore, to ensure the robustness of SR results, it is essential to clearly mandate the conduct and reporting of sensitivity analyses and to recommend that authors systematically describe how key methodological assumptions influence the findings.

Meanwhile, adherence to item 24 (Registration and protocol) was observed in only two of the 20 studies, representing a mere 10% compliance rate. As highlighted in previous research [3], the importance of protocol registration has been repeatedly emphasized. To address this issue, it is necessary to mandate prospective registration of SR and meta-analysis protocols with internationally recognized registries, such as PROSPERO, at the time of submission. Furthermore, considering evidence that recommending the reporting of protocol registration and the availability of data and code contributes to improved reporting quality [4], policy measures that strengthen evaluation criteria for transparency-related items are particularly warranted.

A review over time indicated that adherence to items 12 (Effect measures), 13 (Synthesis methods), 14 (Reporting bias assessment), 20 (Results of syntheses), and 24 (Registration and protocol) showed a marked improvement in studies published in 2024 and later compared to earlier periods. Similarly, reporting of item 27 (Availability of data, code, and other materials) clearly increased from 2023 onwards relative to previous years. These temporal changes are likely closely related to modifications in journal author guidelines and the strengthening of reporting requirements. Similarly, previous studies analyzing SRs using the PRISMA guidelines have reported that while certain items consistently demonstrate 100% adherence, the compliance rates for other items tend to improve over time [20]. Our findings align with these trends. In particular, ongoing monitoring of improvements in items that previously exhibited low adherence—such as certainty of evidence assessment and protocol registration—is necessary to clearly communicate compliance expectations at the time of submission. Furthermore, methodological reviews that continuously track trends in PRISMA 2020 adherence for SRs published in JKBNS will be essential in the future.

In this study, a conceptual mapping was performed between the PRISMA 2020 checklist and the AMSTAR-2 and ROBIS tools to analyze the similarities and differences between reporting guidelines and methodological quality assessment instruments. The results indicated that the introduction and methods-related items of PRISMA 2020 conceptually corresponded closely with the core evaluation items of AMSTAR-2 and domains 1 and 2 of ROBIS. Furthermore, data collection, risk of bias assessment, and synthesis methods were also aligned with key items in AMSTAR-2 and across ROBIS domains. These findings are consistent with recent studies reporting that SRs applying PRISMA 2020 together with AMSTAR-2 and ROBIS exhibit significantly higher quality in terms of reporting completeness, methodological rigor, and risk of bias compared to reviews using a single tool. The concurrent use of these three tools contributes to correcting or mitigating incomplete reporting, methodological shortcomings, and bias risk [21].

Furthermore, while ROBIS remains a traditional framework focused on assessing the inherent risk of bias in SRs, PRISMA 2020 and AMSTAR-2 reflect recent changes in the research environment. This aligns with previous studies indicating that PRISMA 2020, which evaluates reporting quality, AMSTAR-2, which assesses methodological reliability, and ROBIS, which evaluates risk of bias, each play distinct roles and enable complementary analyses [22, 23]. Moreover, recent research emphasizes that relying on a single guideline or assessment tool is insufficient to ensure the reliability and reproducibility of studies, highlighting the need for concurrent application of multiple tools [21]. Such an approach is essential for comprehensively evaluating the various aspects of SRs.

From both research and practical perspectives, the concurrent use of the three tools (PRISMA 2020, AMSTAR-2, and ROBIS) provides a mechanism to ensure the quality of SRs required for clinical practice, policy-making, and health technology assessment, thereby enhancing the reproducibility and confidence of evidence [4]. This underscores the need for management through strengthened editorial policies at the society and journal level. Specifically, requiring submission of the PRISMA checklist as a mandatory document at the time of manuscript submission, or using PRISMA items as evaluation criteria during the peer-review process, could be effective strategies. In addition, implementing PRISMA 2020 workshops and guideline-based training for researchers can further enhance reporting literacy and improve authors’ ability to comprehensively and transparently report SRs.

In conclusion, this study quantitatively analyzed the reporting status of SRs published in the JKBNS based on the international standard PRISMA 2020, providing valuable insights for enhancing the reliability and transparency of future nursing research. This approach allows authors to systematically self-evaluate the completeness and quality of their reporting before submission, while also enabling the editorial board to verify guideline adherence during peer review. In medical journals, ROBIS alone is often sufficient for assessing the risk of bias. However, in nursing research—where interpretation, contextual relevance, and implications for future practice and research are emphasized—ROBIS alone may be insufficient to fully capture reporting quality. Reflecting this perspective, we recommend that journals, including JKBNS, adopt PRISMA 2020 as the primary reporting guideline and require authors to submit a completed PRISMA checklist as supplementary material. This practice is expected to strengthen reporting transparency, enhance the methodological rigor of submitted manuscripts, and support alignment with international standards.

## CONCLUSION

This study evaluated the reporting quality of SRs published in the JKBNS from 2011 to 2024 using the PRISMA 2020 guidelines. Although the overall adherence rate was 64.7%, several essential items—particularly protocol registration, sensitivity analyses, and certainty of evidence—were consistently underreported. Improving the transparency and methodological rigor of SRs requires greater attention to these core components.

To strengthen the reliability and clinical applicability of future SRs, authors should incorporate essential elements such as protocol documentation and comprehensive evidence assessments. Journals, including JKBNS, are encouraged to revise submission guidelines to explicitly require adherence to PRISMA 2020 and to recommend the concurrent use of AMSTAR-2 and ROBIS. Implementing standardized checklists during peer review and manuscript submission may further enhance reporting completeness and elevate the journal's alignment with international standards.

## Figures and Tables

**Table 1.** Characteristics of Systematic Reviews Published in the *Journal of Korean Biological Nursing Science* (2011~2024) (N = 20)

Characteristics		n (%)	M ± SD (range)
Publication year	2011~2015	3 (15.0)	
	2016~2020	10 (50.0)	
	2021~2024	7 (35.0)	
Type of article	SR	13 (65.0)	
	SR with meta-analysis	7 (35.0)	
PICO	No	10 (50.0)	
	Yes	10 (50.0)	
Registration of protocol	No	18 (90.0)	
	Yes	2 (10.0)	
Number of DBs	< 6	9 (45.0)	6.40 ± 3.28 (2~12)
	6~10	7 (35.0)	
	> 10	4 (20.0)	
Type of DB^†^	PubMed	15 (75.0)	
	CINAHL	14 (70.0)	
	Cochrane library	10 (50.0)	
	Embase	10 (50.0)	
	Medline	1 (5.0)	
	PsycINFO	3 (15.0)	
	Google Scholar	3 (15.0)	
	WoS	4 (20.0)	
	Scopus	5 (25.0)	
	RISS	17 (85.0)	
	KISS	9 (45.0)	
	NAL	5 (25.0)	
	DBpia	7 (35.0)	
	KMbase	8 (40.0)	
	KoreaMed	5 (25.0)	
	NDSL	6 (30.0)	
	Others	9 (45.0)	
Data collection (people)	Not reported	2 (10.0)	
	1	1 (5.0)	
	≥2	17 (85.0)	
Number of included studies	≤ 10	8 (40.0)	14.80 ± 10.89 (5~53)
	11~20	8 (40.0)	
	> 20	4 (20.0)	
Effect size	Not reported	13 (65.0)	
	Hedge's g	4 (20.0)	
	SMD or WMD	3 (15.0)	
Heterogeneity^*^	Not reported	13 (65.0)	
	Higgins' I^2^	7 (35.0)	
	Cochran’s Q statistic	3 (15.0)	
Publication bias^*^	Not reported	13 (65.0)	
	Funnel plot	6 (30.0)	
	Trim and fill plot	3 (15.0)	
	Egger's regression test	2 (10.0)	
	Begg's test	1 (5.0)	
	Trim and fill method	1 (5.0)	
Quality appraisal	Not reported	6 (30.0)	
	RoB or RoBANS	7 (35.0)	
	JBI	3 (15.0)	
	NOS	2 (10.0)	
	CASP	1 (5.0)	
	Critical review form	1 (5.0)	

M = Mean; SD = Standard deviation; SR = Systematic review; PICO = Population, intervention, comparison, outcome; DB = Database; CINAHL = Cumulative Index to Nursing and Allied Health Literature; Embase = Excerpta Medica database; PsycINFO = Psychological Information Database; WoS = Web of Science; RISS = Research Information Sharing Service; KISS = Korean Studies Information Service System; NAL = National Assembly Library of Korea; DBpia = Database Platform for Information & Academic Resources; KMbase = Korean Medical Database; KoreaMed = Korean Medical Literature Database; NDSL = National Digital Science Library; SMD = Standardized mean difference; WMD = Weighted mean difference; Begg’s test = Begg and Mazumdar rank correlation test; RoB = Risk of Bias; RoBANS = Risk of Bias Assessment Tool for Non-Randomized Studies; JBI = Joanna Briggs Institute; NOS = Newcastle–Ottawa Scale; CASP = Critical Appraisal Skills Programme.

† Multiple choice.

**Table 2.** PRISMA 2020 Item-level Compliance in Systematic Reviews Published in the *Journal of Korean Biological Nursing Science* (2011~2024)

Domains	Items	1	2	3	4	5	6	7	8	9	10	11	12	13	14	15	16	17	18	19	20	Sum
Title	1. Identify the report as a systematic review.	0	1	1	1	1	1	1	0	0	1	1	0	1	1	1	1	1	1	1	1	16
Abstract	2. See the PRISMA 2020 for abstracts checklist	0.5	0.5	0.5	0.5	0.5	1	0.5	0.5	0.5	1	0.5	1	1	0.5	0.5	0.5	1	1	1	1	14
Introduction	3. Describe the rationale	1	1	1	1	1	1	1	1	1	1	1	1	1	1	1	1	1	1	1	1	20
	4. Objectives of the review	1	1	1	1	1	1	1	1	1	1	1	1	1	1	1	1	1	1	1	1	20
Methods	5. Eligibility criteria.	1	1	1	1	1	1	1	0.5	1	1	1	1	1	1	1	1	1	1	1	1	19.5
	6. Information sources.	1	1	1	1	1	1	1	1	1	1	1	1	1	1	1	1	1	1	1	1	20
	7. Search strategy	1	1	1	1	1	1	1	1	1	1	1	1	1	1	1	1	1	1	1	1	20
	8. Selection process	0.5	1	1	1	1	1	1	1	1	1	1	1	1	1	1	1	1	1	1	1	19.5
	9. Data collection process	1	1	1	1	1	1	1	1	1	1	1	1	1	1	1	1	1	1	1	1	20
	10a. Data items for all outcomes	1	0.5	1	1	1	1	1	1	1	1	1	1	1	0	1	0	1	1	1	1	17.5
	10b. Data items for all other variables	0	0.5	0	0.5	1	1	1	0	1	1	1	0	0	0	0	0	0	1	0	0	8
	11. Study risk of bias assessment	0	1	1	1	1	0	1	0	0	1	1	1	1	1	0	1	1	1	1	1	15
	12. Effect measures	0	0	0	0	0	0	1	0	0	1	0	1	0	0	0	0	1	1	1	1	7
	13a. Synthesis methods for study selection	0	0	0	0	0	0	1	0	0	1	0	0	0	0	0	0	1	1	1	1	6
	13b. Synthesis methods for data preparation	0	0	0	0	0	0	1	0	0	1	0	0	0	0	0	0	0	1	1	1	5
	13c. Synthesis methods for result presentation	0	0	0	0	0	0	1	0	0	1	0	1	0	0	0	0	1	1	1	1	7
	13d. Synthesis methods for data synthesis	0	0	0	0	0	0	1	0	0	1	0	1	0	0	0	0	0	1	1	1	6
	13e. Synthesis methods for exploring heterogeneity	0	0	0	0	0	0	1	0	0	1	0	1	0	0	0	0	1	1	1	1	7
	13f. Synthesis methods for sensitivity analysis	0	0	0	0	0	0	0	0	0	0	0	0	0	0	0	0	0	1	1	1	3
	14. Reporting bias assessment	0	0	0	0	0	0	0	0	0	1	0	1	0	0	0	0	1	1	1	1	6
	15. Certainty assessment	0	0	0	0	0	0	0	0	0	0	0	0	0	0	0	0	0	0	0	0	0
Results	16a. Study selection results	1	1	1	1	1	1	1	1	1	1	1	1	1	1	1	1	1	1	1	1	20
	16b. Study selection exclusions	1	1	1	1	1	1	1	1	1	1	1	1	1	1	1	1	1	1	1	1	20
	17. Study characteristics	1	1	1	1	1	1	1	1	1	1	1	1	1	1	1	1	1	1	1	1	20
	18. Risk of bias in studies	0	0	1	1	0	0	1	0	0	1	1	1	1	1	1	1	1	1	1	1	14
	19. Results of individual studies	1	0	1	1	0	1	1	0	0	1	0	1	0	0	1	0	1	1	1	1	12
	20a. Results of syntheses for study characteristics and risk of bias	0	0	0	0	0	0	1	0	0	1	0	1	0	0	0	0	0	1	1	1	6
	20b. Results of syntheses for statistical syntheses	0	0	0	0	0	0	1	0	0	1	0	1	0	0	0	0	1	1	1	1	7
	20c. Results of syntheses for heterogeneity investigations	0	0	0	0	0	0	1	0	0	1	0	1	0	0	0	0	1	1	1	1	7
	20d. Results of syntheses for sensitivity analyses	0	0	0	0	0	0	0	0	0	0	0	0	0	0	0	0	0	1	1	1	3
	21. Reporting biases	0	0	0	0	0	0	0	0	0	1	0	1	0	0	0	0	0	1	1	1	5
	22. Certainty of evidence	0	0	0	0	0	0	0	0	0	0	0	0	0	0	0	0	0	0	0	0	0
Discussion	23a. Discussion for interpretation of results	1	1	1	1	1	1	1	1	1	1	1	1	1	1	1	1	1	1	1	1	20
	23b. Discussion about the limitations of included evidence	1	1	1	1	0	1	1	1	1	1	1	1	1	1	0	1	0	0	0	1	15
	23c. Discussion about the limitations of the review process	0	0	0	1	0	0	0	0	1	0	1	1	0	1	1	1	1	0	0	1	9
	23d. Discussion for implications for practice, policy, and research	1	1	1	1	1	1	1	1	1	1	1	1	1	1	1	1	1	1	1	1	20
Other Information	24a. Registration and protocol regarding registration information	0	0	0	0	0	0	0	0	0	0	0	0	0	0	0	0	0	0	1	1	2
	24b. Registration and protocol regarding protocol access	0	0	0	0	0	0	0	0	0	0	0	0	0	0	0	0	0	0	1	1	2
	24c. Registration and protocol regarding amendments	0	0	0	0	0	0	0	0	0	0	0	0	0	0	0	0	0	0	0	0	0
	25. Support for funding and sponsors	0	1	1	1	0	1	0	0	0	0	0	0	1	1	1	1	1	1	1	1	12
	26. Competing interests	0	0	0	0	0	1	1	1	1	1	1	1	1	1	1	1	1	1	1	1	15
	27. Availability of data, code, and other materials	0	0	0	0	0	0	0	0	0	0	0	0	0	0	1	1	1	1	1	1	6
Total PRISMA2020 score per study (M±SD)		15	17.5	19.5	21	16.5	20	29.5	15	17.5	32	20.5	29	20	19.5	20.5	20.5	29	35	36	38	23.58 ± 7.34

Numbers 1–20 correspond to the included systematic reviews listed in Appendix 1. PRISMA = Preferred Reporting Items for Systematic Reviews and Meta-Analyses; M = Mean; SD = Standard deviation.

**Table 3.** Compliance Rates for the 27 PRISMA 2020 Checklist Items in Systematic Reviews Published in the *Journal of Korean Biological Nursing Science* (2011~2024)

Domains	Items	1	2	3	4	5	6	7	8	9	10	11	12	13	14	15	16	17	18	19	20	Sum
Title	1. Identify the report as a systematic review.	0	1	1	1	1	1	1	0	0	1	1	0	1	1	1	1	1	1	1	1	16
Abstract	2. See the PRISMA 2020 for abstracts checklist	0.5	0.5	0.5	0.5	0.5	1	0.5	0.5	0.5	1	0.5	1	1	0.5	0.5	0.5	1	1	1	1	14
Introduction	3. Describe the rationale	1	1	1	1	1	1	1	1	1	1	1	1	1	1	1	1	1	1	1	1	20
	4. Objectives of the review	1	1	1	1	1	1	1	1	1	1	1	1	1	1	1	1	1	1	1	1	20
Methods	5. Eligibility criteria.	1	1	1	1	1	1	1	0.5	1	1	1	1	1	1	1	1	1	1	1	1	19.5
	6. Information sources.	1	1	1	1	1	1	1	1	1	1	1	1	1	1	1	1	1	1	1	1	20
	7. Search strategy	1	1	1	1	1	1	1	1	1	1	1	1	1	1	1	1	1	1	1	1	20
	8. Selection process	0.5	1	1	1	1	1	1	1	1	1	1	1	1	1	1	1	1	1	1	1	19.5
	9. Data collection process	1	1	1	1	1	1	1	1	1	1	1	1	1	1	1	1	1	1	1	1	20
	10. Data items	1	1	1	1	1	1	1	1	1	1	1	1	1	0	1	0	1	1	1	1	18
	11. Study risk of bias assessment	0	1	1	1	1	0	1	0	0	1	1	1	1	1	0	1	1	1	1	1	15
	12. Effect measures	0	0	0	0	0	0	1	0	0	1	0	1	0	0	0	0	1	1	1	1	7
	13. Synthesis methods	0	0	0	0	0	0	0.5	0	0	0.5	0	0.5	0	0	0	0	0.5	1	1	1	5
	14. Reporting bias assessment	0	0	0	0	0	0	0	0	0	1	0	1	0	0	0	0	1	1	1	1	6
	15. Certainty assessment	0	0	0	0	0	0	0	0	0	0	0	0	0	0	0	0	0	0	0	0	0
Results	16. Study selection	1	1	1	1	1	1	1	1	1	1	1	1	1	1	1	1	1	1	1	1	20
	17. Study characteristics	1	1	1	1	1	1	1	1	1	1	1	1	1	1	1	1	1	1	1	1	20
	18. Risk of bias in studies	0	0	1	1	0	0	1	0	0	1	1	1	1	1	1	1	1	1	1	1	14
	19. Results of individual studies	1	0	1	1	0	1	1	0	0	1	0	1	0	0	1	0	1	1	1	1	12
	20. Results of syntheses	0	0	0	0	0	0	0.5	0	0	0.5	0	0.5	0	0	0	0	0.5	1	1	1	5
	21. Reporting biases	0	0	0	0	0	0	0	0	0	1	0	1	0	0	0	0	0	1	1	1	5
	22. Certainty of evidence	0	0	0	0	0	0	0	0	0	0	0	0	0	0	0	0	0	0	0	0	0
Discussion	23. Discussion	1	1	1	1	0.5	1	1	1	1	1	1	1	1	1	1	1	1	0.5	0.5	1	18.5
Other Information	24. Registration and protocol	0	0	0	0	0	0	0	0	0	0	0	0	0	0	0	0	0	0	1	1	2
	25. Support for funding and sponsors	0	1	1	1	0	1	0	0	0	0	0	0	1	1	1	1	1	1	1	1	12
	26. Competing interests	0	0	0	0	0	1	1	1	1	1	1	1	1	1	1	1	1	1	1	1	15
	27. Availability of data, code, and other materials	0	0	0	0	0	0	0	0	0	0	0	0	0	0	1	1	1	1	1	1	6
Total PRISMA2020 score per study (M±SD)		12	14.5	16.5	16.5	13	16	18.5	12	12.5	21	15.5	20	17	15.5	17.5	16.5	22	23.5	24.5	25	17.48 ± 4.03
PRISMA 2020 compliance rates (%)		44.4	53.7	61.1	61.1	48.1	59.3	68.5	44.4	46.3	77.8	57.4	74.1	63.0	57.4	64.8	61.1	81.5	87.0	90.7	92.6	64.7 ± 14.9

Numbers 1–20 correspond to the included systematic reviews listed in Appendix 1. PRISMA = Preferred Reporting Items for Systematic Reviews and Meta-Analyses; M = Mean; SD = Standard deviation.

**Table 4.** Item-level Mapping among PRISMA 2020, AMSTAR-2, and ROBIS

Domains	Items	AMSTAR-2	ROBIS
Title	1. Identify the report as a systematic review.		
Abstract	2. See the PRISMA 2020 for abstracts checklist	1	
Introduction	3. Describe the rationale		Domain 1.1
	4. Objectives of the review	1	Domain 1.1
Methods	5. Eligibility criteria.	3, 8	Domain 1.2~1.5
	6. Information sources.	4	Domain 2.1~2.2
	7. Search strategy	4	Domain 2.3~2.4
	8. Selection process	5	Domain 2.5
	9. Data collection process	6	Domain 3.1
	10a. Data items for all outcomes	8	Domain 3.3
	10b. Data items for all other variables	8	Domain 3.3
	11. Study risk of bias assessment	9	Domain 3.4~3.5
	12. Effect measures	11	Domain 4.3
	13a. Synthesis methods for study selection	11	Domain 4.1
	13b. Synthesis methods for data preparation	11	Domain 4
	13c. Synthesis methods for result presentation	11	Domain 4
	13d. Synthesis methods for data synthesis	11	Domain 4.3
	13e. Synthesis methods for exploring heterogeneity	11	Domain 4.3
	13f. Synthesis methods for sensitivity analysis	11	Domain 4.5
	14. Reporting bias assessment	15	Domain 4.5
	15. Certainty assessment	13	Domain 4.5~4.6
Results	16a. Study selection results	7	Domain 2.1~2.2
	16b. Study selection exclusions	7	
	17. Study characteristics	8	Domain 3.2
	18. Risk of bias in studies	9	Domain 3.4
	19. Results of individual studies	11	Domain 4.3
	20a. Results of syntheses for study characteristics and risk of bias	11, 14	Domain 4.3
	20b. Results of syntheses for statistical syntheses	11	Domain 4.3
	20c. Results of syntheses for heterogeneity investigations	11	Domain 4.3
	20d. Results of syntheses for sensitivity analyses	14	Domain 4.5
	21. Reporting biases	15	Domain 4.5
	22. Certainty of evidence	12, 13	Domain 4.6
Discussion	23a. Discussion for interpretation of results	14	
	23b. Discussion for limitations of included evidence	13, 14	
	23c. Discussion for limitations of review process	14	
	23d. Discussion for implications for practice, policy, and research	14	
Other Information	24a. Registration and protocol regarding registration information	2	
	24b. Registration and protocol regarding protocol access	2	
	24c. Registration and protocol regarding amendments	2	
	25. Support for funding and sponsors	10, 16	
	26. Competing interests	16	
	27. Availability of data, code, and other materials		

PRISMA = Preferred Reporting Items for Systematic Reviews and Meta-Analyses; AMSTAR-2 = A Measurement Tool to Assess Systematic Reviews 2; ROBIS = Risk of Bias in Systematic Reviews.

## Data Availability

All data extracted from published articles are available upon reasonable request.

## Appendix 1.

A1. Cha YR, Won SJ. An analysis of aromatherapy intervention studies in nursing. Journal of Korean Biological Nursing Science. 2013;15(2)54–64. https://doi.org/10.7586/jkbns.2013.15.2.54

A2. Jeong SH. Systematic review of the literatures on music intervention for neurological patients in Korea. Journal of Korean Biological Nursing Science. 2013;15(2):65–73. https://doi.org/10.7586/jkbns.2013.15.2.65

A3. Kim H, Chang SJ. Clinical usefulness of gastric residual volume as an indicator to provide approximately enteral nutrition for patients in intensive care units: a systematic literature review. Journal of Korean Biological Nursing Science. 2014;16(4):267–275. https://doi.org/10.7586/jkbns.2014.16.4.267

A4. Kim SH, Lee YJ, Lee SY, Chu SH. Current interventions to improve adherence to immunosuppressants in liver transplant recipients: a systematic review. Journal of Korean Biological Nursing Science. 2016;18(1):17–26. https://doi.org/10.7586/jkbns.2016.18.1.17

A5. Kim JH, Kwon SB, Kim HJ, Choi GH, Lee HM. Effects of horticultural therapy for the korean elderly: a systematic literature review. Journal of Korean Biological Nursing Science. 2016;18(3):153–159. https://doi.org/10.7586/jkbns.2016.18.3.153

A6. Ahn S, Choi S, Jung HJ, Chu SH. Current pharmacogenetic approach for oxaliplatin-induced peripheral neuropathy among patients with colorectal cancer: a systematic review. Journal of Korean Biological Nursing Science. 2018;20(2):55–66. https://doi.org/10.7586/jkbns.2018.20.2.55

A7. Hyun JS, Kim EJ, Han JH, Kim NH. Effects of simulation-based education for emergency patient nursing care in Korea: a meta analysis. Journal of Korean Biological Nursing Science. 2019;21(1):1–11. https://doi.org/10.7586/jkbns.2019.21.1.1

A8. Jeon HK, Yoo HY. Analysis of obesity intervention programs in adolescents: focused on endothelium functions. Journal of Korean Biological Nursing Science. 2019;21(2):99–107. https://doi.org/10.7586/jkbns.2019.21.2.99

A9. Lee SN. A review on the measurement variables of nursing research for patients with head and neck cancer in Korea. Journal of Korean Biological Nursing Science. 2019;21(3):161–168. https://doi.org/10.7586/jkbns.2019.21.3.161

A10. Kang P, Kim J, Kim MJ. Effect of vitamin D on muscular strength in postmenopausal women: a meta-analysis. Journal of Korean Biological Nursing Science. 2019;21(4):239–248. https://doi.org/10.7586/jkbns.2019.21.4.239

A11. Noh EY, Cho Y, Lee Y, Yun S. A systematic review focused on health behavior and physiological indicators of diabetic patients in interventional studies based on health belief model. Journal of Korean Biological Nursing Science. 2020;22(1):1–10. https://doi.org/10.7586/jkbns.2020.22.1.1

A12. Chae YR, Lee SH. Effect of aerobic exercise on serum lipids, weight and body mass index in patients with hypertension: a systematic review and meta-analysis. Journal of Korean Biological Nursing Science. 2020;22(1):11–22. https://doi.org/10.7586/jkbns.2020.22.1.11

A13. Chae YR, Lee SH. Systematic review of forest therapy program for adult patients with diseases. Journal of Korean Biological Nursing Science. 2020;22(3):157–171. https://doi.org/10.7586/jkbns.2020.22.3.157

A14. Oh J, Kim M, Chu SH. Role of oxytocin in post-traumatic stress disorder: a systematic review. Journal of Korean Biological Nursing Science. 2022;24(1):1–16. https://doi.org/10.7586/jkbns.2022.24.1.1

A15. Shin JW, Yu MY, Son YJ. Frailty assessed by the electronic frailty index and its impact on health outcomes in older adults with chronic diseases: a systematic review. Journal of Korean Biological Nursing Science. 2023;25(4):229–242. https://doi.org/10.7586/jkbns.23.0015

A16. Gudisa GG, Jun S. Adherence to antiretroviral therapy and associated factors among HIV-positive adolescents in Sub-Saharan Africa: a systematic review. Journal of Korean Biological Nursing Science. 2023;25(4):266–275. https://doi.org/10.7586/jkbns.23.0020

A17. Kim DY, Jeon MY, Eun Y, Jeong DI. Exercise and adults with hemophilia: a systematic review and meta-analysis. Journal of Korean Biological Nursing Science. 2024;26(1):1–15. https://doi.org/10.7586/jkbns.23.022

A18. Cho MK, Kim MY. The effect of aromatherapy on pain in individuals with diabetes: a systematic review and meta-analysis. Journal of Korean Biological Nursing Science. 2024;26(2):71–82. https://doi.org/10.7586/jkbns.24.012

A19. Cho MK, Kim MY. Can aromatherapy reduce restless legs syndrome in hemodialysis patients? A systematic review and meta-analysis. Journal of Korean Biological Nursing Science. 2024;26(3):163–176. https://doi.org/10.7586/jkbns.24.022

A20. Cho MK, Kim MY. The effects of ear acupressure therapy on obstetric and gynecological pain in women: a systematic review and meta analysis. Journal of Korean Biological Nursing Science. 2024;26(4):271–287. https://doi.org/10.7586/jkbns.24.035
